# Hypothyroidism and Sturge-Weber Syndrome associated with Bilateral Port-wine Nevus

**DOI:** 10.5005/jp-journals-10005-1339

**Published:** 2016-04-22

**Authors:** Gyanendra Saroj, Anshul Gangwar, Jatinder Kaur Dhillon

**Affiliations:** 1 Associate Professor, Department of Pedodontics and Preventive Dentistry Maulana Azad Institute of Dental Sciences, New Delhi, India; 2 Professor, Department of Pedodontics and Preventive Dentistry, Institute of Dental Sciences, Bareilly, Uttar Pradesh, India; 3 Assistant Professor, Department of Pedodontics and Preventive Dentistry Maulana Azad Institute of Dental Sciences, New Delhi, India

**Keywords:** Hypothyroidism, PWSs, Sturge-Weber syndrome.

## Abstract

Sturge-Weber syndrome (SWS) is a rare, nonhereditary developmental condition that is characterized by a hamartomatous vascular proliferation of the brain, resulting in multiple angiomas that occur on the same side due to arteriovenous malformations. It is believed to be caused by persistence of a vascular plexus around the cephalic portion of the neural tube and is present at birth in about 1 in every 50,000 babies.

It is one of the phakomatoses which is often associated with port-wine stains (PWSs) of the face, glaucoma, seizures, mental retardation and ipsilateral leptomeningeal angioma. Many people with SWS probably never know they have it. Hypothyroidism is a condition that arises from inadequate release of thyroid-stimulating hormone to stimulate an otherwise normal thyroid gland. This condition is often associated with a deficient secretion of other pituitary hormone, and growth hormone deficiency occurs with an increased prevalence in SWS, presumably secondary to involvement of the hypothalamic-pituitary axis. Diagnosis is made by the presence of a facial PWS and evidence of leptomeningeal angioma either by skull X-ray or computed tomography scan that shows intracranial calcifications. Presently, there is no specific treatment for SWS, and the management of the clinical manifestations and complications is still far from adequate.

Here, we report the case of hypothyroidism associated with SWS with oral and facial manifestations in an 11-year-old boy.

**How to cite this article:** Saroj G, Gangwar A, Dhillon JK. Hypothyroidism and Sturge-Weber Syndrome associated with Bilateral Port-wine Nevus. Int J Clin Pediatr Dent 2016;9(1): 82-85.

## INTRODUCTION

Sturge-Weber syndrome (SWS) or encephalotrigeminal angiomatosis is a rare congenital neurological and skin disorder. Sturge-Weber syndrome was initially described by Schirmer, in 1860, and was then specified by Sturge,^1^ who associated the dermatological and ophthalmic changes to the disease’s neurological manifestations. In 1992, Weber complemented it through the documentation of these patients’ cerebral radiologic alterations.

Sturge-Weber syndrome is an embryonal developmental anomaly, resulting from errors in mesodermal and ectodermal development. Unlike other neurocuta-neous disorders (phakomatoses), SWS occurs sporadically (i.e., does not have a hereditary etiology). It is caused by a somatic activating mutation occurring in the Guanine Nucleotide Binding Protein (G Protein), Q Polypeptide gene and persistence of vascular plexus round the cephalic portion of the neural tube. This plexus develops during the 6th week of intrauterine development but normally undergoes regression during the 9th week.^2^

Sturge-Weber syndrome is a sporadic disorder that occurs with frequency of 1:50,000 births. Males are affected more and no racial predilection has been observed.

The predominant clinical manifestations of SWS include cutaneous manifestations like unilateral cutaneous vascular nevus (following the ophthalmic and maxillary division of the 5th cranial nerve) and neurological manifestations including progressive seizures, ipsilateral glaucoma, hemiatrophy, contralateral hemiparesis, hemianopia, and mental retardation.

Oral changes are noted in 40% of cases in the form of angiomatosis of lips causing macrocheilia, resulting in hemiatrophy of buccal mucosa, floor of mouth, and palatal mucosa. Other abnormalities reported are severe gingival enlargement, pyogenic granulomas, unilateral alveolar hypertrophy, ipsilateral premature or delayed eruption, and malocclusion.^3^ Intracranial convolutional calcification is discernible on skull radiograph.^4^

Most potent vascular lesions will blanch under pressure. Large lesions are warm and may even be pulsatile if associated with a large vessel. The blood vessels have simple endothelial lining and connective tissue stroma. Such lesions characteristically bleed profusely when traumatized.

This syndrome is of rare occurrence and management becomes complicated due to risk of hemorrhage. This report illustrates the case of SWS in an 11-year-old boy.

## CASE REPORT

An 11-year-old boy reported to the Department of Pedo-dontics, with the chief complaint of gum enlargement and bleeding while brushing and bluish red discoloration on the face.

The patient’s mother reported that the bluish red discoloration has been present on his face since birth and it has gradually darkened with age.

**Fig. 1:**
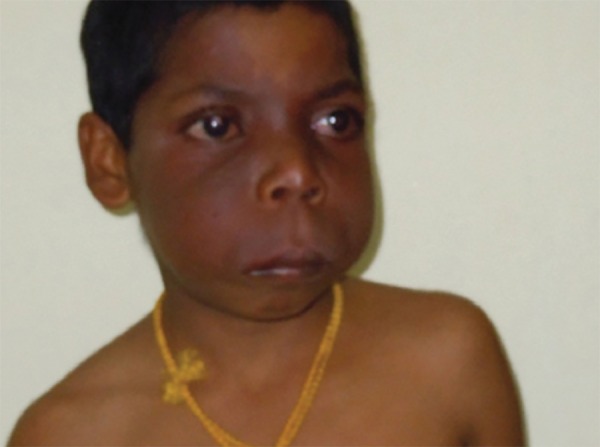
General appearance

None of the family members had similar abnormalities but his family history was significant for hypothyroidism in both his maternal and paternal grandmother. The patient had a positive history for convulsions. The last episode took place one month earlier. He had not taken any medicines for his convulsions. The patient presented with right-sided hemiparesis (Fig. 1) and an intelligence quotient below normal (verbal intelligence quotient, 51).

**Fig. 2:**
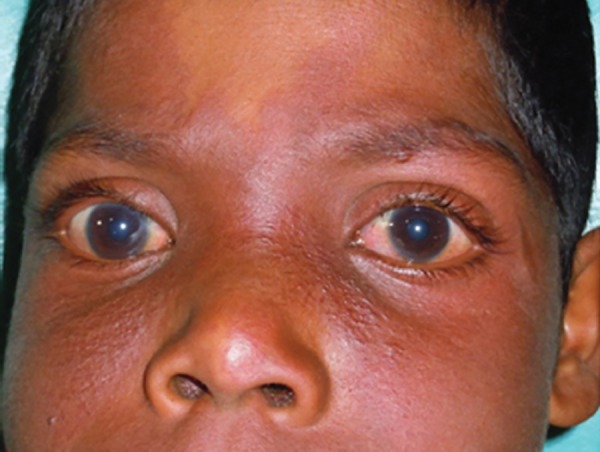
Extraoral appearance

Extraoral examination revealed port-wine stain (PWS) or nevus with bilateral distribution along multiple segments of trigeminal nerve since birth. It extended from the middle of the forehead and involved the eye, nose, cheek, philtrum. When digital pressure was applied on the PWS blanching was readily identified (Fig. 2).

**Fig. 3:**
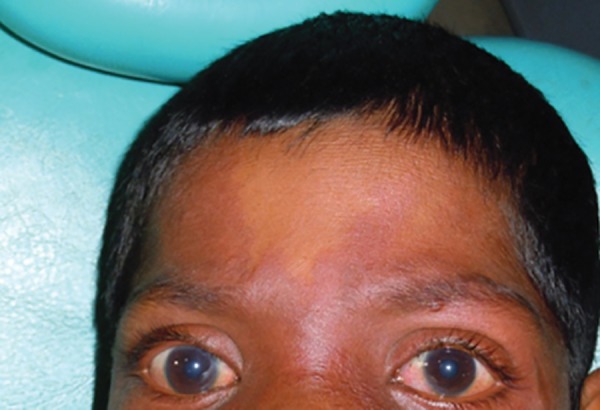
Dilated vessels in eyes

Examination of the eyes revealed dilated blood vessels in both the eyes, greatest risk of glaucoma, and therefore the patient was advised ophthalmic consultation (Fig. 3).

**Fig. 4:**
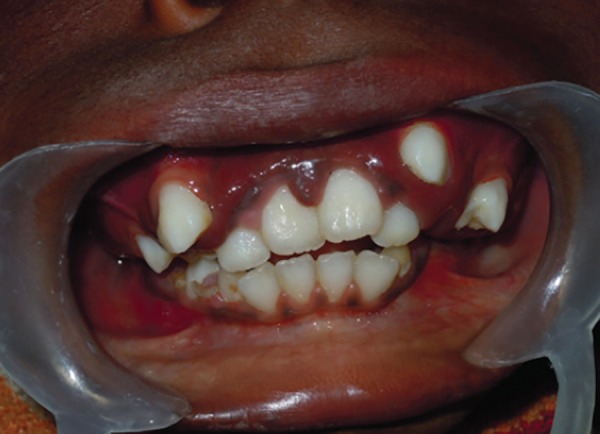
Intraoral frontal view

Intraoral examination revealed reddish-pink gingival overgrowth involving labial and palatal gingiva and loss of knife edge contour. There was slight blanching of the gingival enlargement on pressure, raising concerns that it might be an angiomatosis (vascular) nature of gingival enlargement. There was history of spontaneous bleeding and also on brushing and slight provocation (Fig. 4).

Thyroid function tests were performed, which revealed free thyroxine at 0.62 ng/dl (normal range, 0.7-1.6 ng/dl); total free thyroxine at 4.5 μg/dl (normal range, 5.5-11.0 μg/dl); thyroid-stimulating hormone at 2.69 IU/ml (normal range, 0.47-4.68 IU/ml). The results of his laboratory studies were consistent with hypothyroidism.

Tomographic examination was carried out and showed the presence of thin meningeal gyri calcifications.

**Fig. 5:**
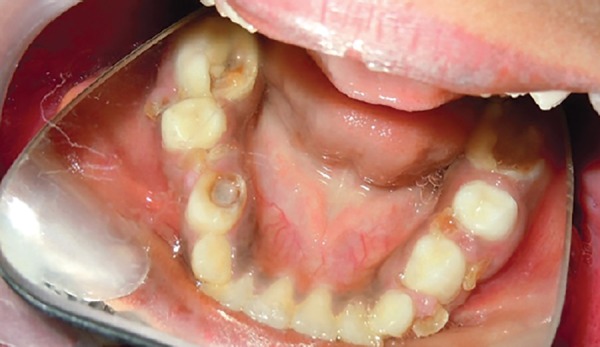
Occlusal view

Physician’s consent was taken prior to treatment. The proposed dental treatments were removal of root stumps, the left mandibular first and second deciduous molar, right first deciduous molar and root canal treatment of permanent molars. A thorough plaque control regimen was started, including oral prophylaxis at regular intervals, oral hygiene instructions and motivation of the patient at each visit (Fig. 5).

## DISCUSSION

Sturge-Weber syndrome is a sporadic neurocutaneous disease, also known as Sturge-Weber disease, encepha-lotrigeminal angiomatosis, meningofacial angiomatosis and Sturge-Weber-Dimitri syndrome, characterized by focal PWS, ocular abnormalities (glaucoma) and choroidal hemangioma and leptomeningeal angioma, most often involving the occipital and parietal lobes.^5^

Port-wine stain represents hamartous capillary malformation and is named so due to deep red hue that they leave on the skin or mucosa.^6^

This syndrome consists of constellation of clinical features like facial nevus, seizures, hemiparesis, intracranial calcifications (termed "tramline", "tram-trac" or "trolley track" calcifications) and mental retardation.^7^

Sturge-Weber syndrome has extremely varied clinical features and its diagnosis can be characterized by PWS on the face followed by other signs, such as glaucoma, epilepsy and mental retardation.

The port-wine nevus is localized in the face, especially on the right side of the face, and is detected in 87-90% of the cases. The lesion extension over the midline is observed in 50% of the patients and bilateral involvement in 33% of the cases.^8^

Port-wine stains in childhood are classically faint, pink macules that tend to darken progressively to red purple, may be isolated with well-delineated border or may be very diffuse. It has usually unilateral distribution along with one or more segments of trigeminal nerve. Occasionally bilateral involvement or lesion on other parts of the body may occur.^2^

Not all patients with facial PWSs have Sturge-Weber angiomatosis. Only patients with involvement along the distribution of the ophthalmic branch of trigeminal nerve are at risk for the development of full condition.

Sturge-Weber syndrome is referred to as complete when both central nervous system and facial angiomas are present and incomplete when only one area is affected without the other. In 1992, Roach categorized SWS variants into three types^9^:

1. Type I (Classic) - This is the most commonly described form, with both facial and leptomeningeal angiomas. Seizures usually occur in the first year of life, and ocular involvement, most commonly glaucoma, is likely to be present.
2. Type II - This type manifests with facial angioma and the possibility of glaucoma, but with no evidence of intracranial disease.
3. Type III (Forme fruste) - This type only involves leptomeningealangioma, with no facial nevus and usually no ocular manifestation of glaucoma.

In both types II and III SWS, the natural course of the disease and progression over time is not known completely and additional research is necessary.

When PWS involves the eyelid, vascular abnormalities of the conjunctiva, episclera, retina and choroid may occur. Patients with the most severe anomalies in conjunctival and episcleral vessels are at greatest risk of glaucoma, and overall, 30 to 70% of patients with SWS will develop glaucoma. As with intracranial involvement, the presence of a cutaneous vascular malformation in the distribution of the ophthalmic division of the trigeminal nerve increases the probability of glaucoma.^10^

Regular eye examinations and a high index of suspicion between appointments are crucial for these individuals. Even for children without glaucoma, regular ophthalmologic surveillance is necessary until adulthood as some may develop raised intraocular pressure much later. In rare instances, the glaucoma could develop as late as the 4th decade.

Differential diagnosis of SWS includes Klippel-Trenaunay-Weber syndrome, hereditary hemorrhagic telangiectasia (Osler-Weber-Rendu disease), Maffucci syndrome, Von Hippel-Lindau disease.^6^

As seen in the present case, the 11-year-old child had bilateral facial PWS since birth. The child was also mentally retarded and had a history of seizures, which may be considered a case of complete SWS, but according to Roach classification, it may be type I.

MedSearch database revealed that 7% of patients with diagnosed SWS and brain involvement reported maternal hypothyroidism. The type of maternal hypo-thyroidism is not known in these data. The prevalence of hypothyroidism in women in the general population ranges between 1 and 12%. Therefore, limited data do not support a role for maternal hypothyroidism as a risk factor for SWS.

Our patient was diagnosed as a result of routine checkups of thyroid function. However, it is crucial to keep in mind that patients with SWS have an increased prevalence of growth hormone deficiency, thereby causing the disruption of the hypothalamic-pituitary axis by SWS. Hence, the pituitary axis must be evaluated if there are any suggestive clinical signs and also routine thyroid function test.

Treatment and prognosis depend upon the nature and severity of clinical features. Besides this, the most important prognostic factor is the absence or presence of seizures and if present the age of onset of the seizures.

Treatment is aimed at seizure control, prevention and treatment of glaucoma, recognition and management of emotional and behavior problems with medication, psychotherapy and behavioral therapies, symptomatic treatment of headaches and cosmetic attention to PWS. Physical and occupational therapy may also be beneficial. No preventive measures are known.

The presence of PWS can cause deep psychological trauma to the patient and development of personality is affected. Port-wine stains can be improved by high dose of hydrocortisone, dermabrasion, tattooing and flash lamp pulsed dye lasers and cryotherapy or camouflaged with makeup.

The patient was referred to a dermatologist for his cosmetic treatment of PWSs. But the patient’s family did not show interest in the treatment as they were poor and could not afford the cost of the treatment.

In the present case, gingival overgrowth was managed by oral hygiene maintenance, but despite stringent plaque control, in some cases gingivectomy may be required. Combination of gingivectomy and laser can be used if the overgrowth is very large.

In these cases, achieving hemostasis can be a significant problem. Various methods used to manage the risk of hemorrhage are use of hemostatic agent, provision for blood transfusion, use of postoperative splints, neodymium-doped yttrium aluminium garnet (Nd:YAG) lasers or injecting sclerosing solutions.

Poor oral hygiene can lead to secondary inflammatory gingival enlargement and high decayed-missing-filled teeth (DMFT) score. Therefore, patient and parent education along with plaque control measures should be strictly followed to minimize or to prevent these problems.

## CONCLUSION

It is difficult to reach an early diagnosis due to the varied clinical presentations and multifactorial tendency of SWS. The patients with oral changes must be subjected to oral cavity examinations periodically to look for and prevent any complication. Extra care must be taken when performing surgical procedure in the affected areas of the mouth. The great frequency of oral manifestations of SWS demands dentists’ knowledge of this syndrome, clinical features and treatment modalities.

## References

[1] Zhou J, Li NY, Zhou XJ, Wang JD, Ma HH, Zhang RS (2010). Sturge-Weber syndrome: a case report and review of literatures. Chin Med J.

[2] Neville BM., Damm DD., Allen CM., Bouquot JL (2009). Oral and maxillofacial pathology..

[3] Mukhopadhyay S (2008). Sturge-Weber syndrome: a case report. J Indian Soc Pedod Prev Dent.

[4] Lynch MA., Brightman VJ., Greenberg MS (1998). Burket’s oral medicine: diagnosis and treatment..

[5] Khan HI, Afzal MF, Anjum F, Javed T (2006). Sturge-Weber syndrome. A case report. J Pak Assoc Dermatologists.

[6] Fishman SJ, Mulliken JB (1993). Hemangiomas and vascular malformations of infancy and childhood. Pediatr Clin North Am.

[7] Chitlangia M, Parakh P, Yadav S, Shah GS, Mishra OP (2012). Sturge-Weber syndrome with bilateral port-wine nevus. J Clin Case Rep.

[8] Kalakonda B, Pradeep K, Mishra A, Reddy K, Muralikrishna T, Lakshmi V, Challa R (2013). Periodontal management of Sturge-Weber syndrome. Case Rep Dentistry.

[9] Roach ES (1992). Neurocutaneous syndromes. Pediatr Clin North Am.

[10] Maria BL, Olson LL., Comi AM (2005). Sturge-Weber syndrome: current management in child neurology..

